# Support Immersion Endoscopy in Post-Extraction Alveolar Bone Chambers: A New Window for Microscopic Bone Imaging *In Vivo*

**DOI:** 10.1371/journal.pone.0145767

**Published:** 2015-12-29

**Authors:** Wilfried Engelke, Marcio Lazzarini, Walter Stühmer, Víctor Beltrán

**Affiliations:** 1 Department Oral and Maxillofacial Surgery, Georg-August-University Hospital, Göttingen, Germany; 2 Department of Molecular Biology of Neuronal Signals, Max Planck Institute of Experimental Medicine, Göttingen, Germany; 3 Research Centre in Dental Sciences (CICO), Dental School, Universidad de La Frontera, Temuco, Chile; University of Zaragoza, SPAIN

## Abstract

Using an endoscopic approach, small intraoral bone chambers, which are routinely obtained during tooth extraction and implantation, provide visual in vivo access to internal bone structures. The aim of the present paper is to present a new method to quantify bone microstructure and vascularisation in vivo. Ten extraction sockets and 6 implant sites in 14 patients (6 men / 8 women) were examined by support immersion endoscopy (SIE). After tooth extraction or implant site preparation, microscopic bone analysis (MBA) was performed using short distance SIE video sequences of representative bone areas for off-line analysis with ImageJ. Quantitative assessment of the microstructure and vascularisation of the bone in dental extraction and implant sites in vivo was performed using ImageJ. MBA revealed bone morphology details such as unmineralised and mineralised areas, vascular canals and the presence of bleeding through vascular canals. Morphometric examination revealed that there was more unmineralised bone and less vascular canal area in the implant sites than in the extraction sockets.

## Introduction

Microscopic observation of vital bone has been a challenging task. Windows to the bone tissue in animal experiments were first opened by P.I. Branemark. Orthotopic or “bone” chambers were developed by Branemark for viewing microcirculation at and near medullary hematopoietic sites [1]. He made the first *in vivo* observations of microcirculation in medullary sinusoids and endosteal vessels, noting that their blood velocities were comparable to those reported from nonosseous tissues [2].

Uncovering the details of microvascular physiology has been successful, largely because of the application of intra-vital microscopy to an increasing variety of tissues [3]. Hsieh A *et al*. (2001) [4] used a model for critical limb ischaemia by occluding femoral vessels in rabbits and observing cortical bone *in vivo* with an implanted tibial window, which included an optical bone chamber implant with intravital microscopy. Desmons et al. (2010) [5] evaluated bone vascular parameters using an optical bone chamber implanted onto the calvaria of rabbits following X-ray irradiation. A computer-based semi-automatic system was described to quantify superficial bone vascular network parameters. Brown *et al*. (2010) [6] reported the use of *in vivo* light microscopy for soft tissue and stated that due to limited light penetration, epifluorescence and confocal microscopy are typically limited to the outer 50–100 microns of the accessible tissue. Villa et al. (2013) [7] described a method for visualising *in vivo* bone formation within a cell scaffold tissue-engineered construct at a single-cell resolution in three dimensions using two-photon microscopy to visualise osteogenesis. A clinical *in vivo* assessment of bone circulation using osteoscopy was reported in orthopaedic surgery for the assessment of the blood supply of the femoral head. Nyarady et al. (2012) [8] reported a technique to determine the relationship between the arterial pressure and perfusion of the femoral head in animal experiments and in humans. They used endoscopes and a mortise-sleeve-optic system connected to a manometer and a saline reservoir to form a closed system. Three categories of bleeding were determined, and in humans, different qualities of femoral head circulation could be observed. Endoscopes for intraosseous surgery have been used for the removal of a bone cyst of the proximal femur [9], and they have also been used during hand surgery [10]. However, endoscopic imaging has not been used on a microscopic level to evaluate bone wall structures.

In oral surgery, different types of endoscopes have been developed and applied for the precise intraoperative examination of alveolar bone structures [11–16]. SIE based on rigid 1.9-mm scopes, in conjunction with a support and irrigation sheath, provides insight into the bone cavity following the implant drill sequence and allows oral surgeons to obtain a direct view of the bone site before placing the implant. This visualisation helps ensure that there are appropriate mechanical and biological conditions for implant placement [11, 15]. Due to the fact that tooth extraction is the most frequent surgical intervention in human bone, this method provides a unique opportunity to obtain data about vascularisation, blood flow parameters and a variety of bone pathologies *in vivo*. Therefore, the aim of the study is to present a method to quantitatively assess the microstructure and vascularisation of bone in dental extraction sites *in vivo*.

## Patients and Methods

SIE videos with high quality and resolution were selected from surgeries performed in the Center of Oral and Maxillofacial Surgery at the University of Göttingen between 1998 and 2012. The retrospective analysis of patients was approved by the committee of the Faculty of Medicine (Ethikkommission der Medizinische Fakultät, N 25/9/13), University of Göttingen and the all data provided to researchers were anonymised to ensure that individual patients cannot be identified. SIE was performed immediately after flapless tooth extraction or at flapless or miniflap implant sites. The videos of 9 patients were selected for alveolar analysis (3 men / 6 women aged from 26 to 83 years old) for a total of 10 alveolus sites and of 5 patients for implant sites (1 men / 4 women aged from 18 to 76 years old) for a total of 6 alveolus sites.

### 3.1. Short Distance Support Immersion Alveoloscopy (SD-SIE)

The endoscopic equipment consisted of rigid Storz-Hopkins endoscopes with a 1.9-mm diameter with an incorporated metallic support and irrigation tubes (Karl Storz, Tuttlingen, Germany). For SIE, an endoscope with a 1.9-mm diameter and a 30 and 70 degree view angle was used with continuous irrigation via the irrigation tube. SIE allowed for short distance observations with direct contact of the scope to the surgical site. The endoscopes were coupled to a Storz examination unit B 487 and xenon light source with a 300 W capacity of 6000 K. (Karl Storz, Tuttlingen, Germany). In SD-SIE, the scope window was placed as near as possible to the bone surface within a natural chamber formed by the extraction socket or the implant cavity. Observations routinely took place at the deepest aspect of the cavity. Manual jet stream irrigation served to clean the bone surface, and the irrigation flow was stopped immediately before observation; thus, a bone surface could be observed without distortion through the irrigation medium (saline solution).

### 3.2. Microscopic Bone Imaging analysis (MBI)

**Fig 1:**
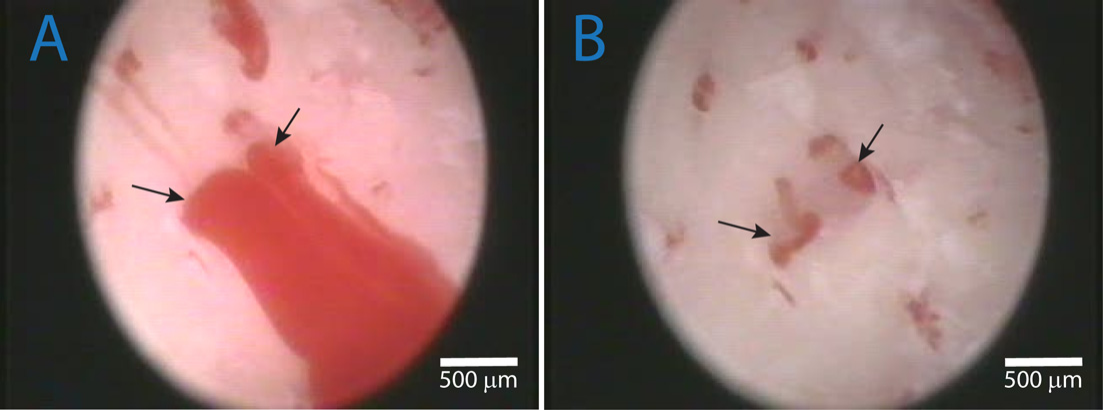
A and B: Identification of vascular canals. Bleeding through vascular canals (black arrow), and SIE without irrigation flow. B: SIE of the same area following intermittent high flow irrigation by saline. The area of the vascular channels is clearly visible.

**Fig 2:**
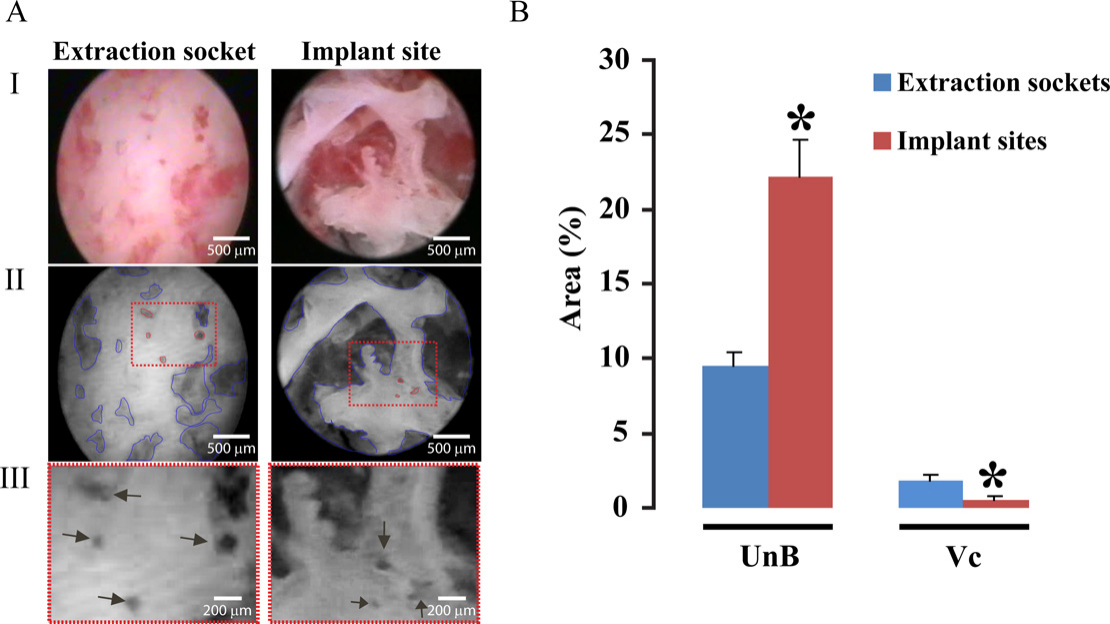
A: Analysis of bone microstructure. Analysis of SIE images in typical extraction (left, extraction socket) and implant sites (right). Original screenshots of an SIE evaluation recorded on video. For better visualisation of unmineralised bone (blue marks) and vascular canals, the images were converted to grey scale, the contrast was increased and the brightness was decreased (AII). The inserts represent the magnification of the vascular canals (black arrows, AIII). B: Quantitative analysis of the unmineralised bone (UnB) and vascular canal (Vc) areas as a percentage of the total area. Implant sites showed more unmineralised bone area than did the extraction sockets (independent t test; p<0.05). The vascular canal area was smaller in implant sites than in extraction socket (Mann-Whitney Test; p<0.05).

Bone analysis was performed in three main steps: 1-endoscopic procedure in vivo, 2-off-line image selection and 3-image analysis (as summarised in Table 1). To obtain high image quality from SIE *in vivo*, six procedures were adopted, including: 1- general view of the bone surface to make the best selection of the area of interest (Aol, 2); 3-high flow (HF) irrigation to remove, for example, the excess of blood and other tissue; 4-observation of the bone area (Aol) and record. SIE *in vivo* recording was performed two times to be sure that the AoI had the best quality (procedure 5 and 6, Table 1). Off-line image selection was performed by 1-AoI in a timeline from the acquisition of SIE *in vivo*; 2-checking the bleeding of structures and 3-selecting the AoI with the minimal bleeding surface frame. This step is mandatory to define the vascular canals (Fig 1 and S1 Movie). The image was then captured (4). Cross checking and repetition were performed to be sure of the vascular canals that were selected (procedure 5 and 6, Table 1). The image analysis from off-line image selection was performed by ImageJ software. The images were transformed in grey scale with a gain of contrast and reduced brightness (1) to improve the definition of the AoI (Fig 2 and Table 1). The freehand selection tool was used in ImageJ to determine the non-mineralised bone areas (3). Vascular canals (4) were identified by observing the original movies that were recorded by the endoscopic procedure *in vivo*. The percentages of unmineralised areas or canal areas were calculated by: (unmineralised) or (total canal area) multiplied by 100 and then divided by the total area selected (procedure 5 and 6, Table 1).

**Table 1:** Procedure of Microscopic Bone Imaging Analysis.

Endoscopic procedure in vivo	Off-line Image Selection	Image Analysis
1) SIE general view of the bone surface (large distance—SIE)	1) Selection of the AoI in a timeline	1) Optimisation of the contrast and brightness
2) Selection of Aol (minimal distance—SIE)	2) Checking the bleeding structures	2) Definition of the total AoI
3) HF Irrigation	3) Selection of the minimal bleeding surface frame	3) Manual identification of the non—mineralised areas
4) Observation of the AoI and record	4) Image capture	4) Identification of the vascular canals areas (Fig 1A and 1B and S1 Movie)
5) HF Irrigation	5) Cross check (effect of irrigation)	5) Calculation of the areas
6) Repetition of the Observation of the AoI and recording	6) Repetition (if necessary)	6) Final report

### 3.3. Data acquisition and statistical analysis

Unmineralised bone and vascular canals were contoured, and the areas were calculated. These areas were normalised by the percentage of the total area selected. A Shapiro-Wilk´s test (p>0.05) [17,18] showed that the data are normally distributed for unmineralised bone, but not for vascular canals. Therefore, the independent t test for parametric values was used to compare the unmineralised bone, and the Mann-Whitney test was used for vascular canals. The level of significance was set at p<0.05. Statistical analysis was performed using SPSS, Inc., software.

## Results

### 4.1. Quantification of unmineralised bone and vascular canals area by SIE in extraction sockets and implant sites

MBI applied in extraction sockets and artificial bone surfaces *in vivo* allowed for the quantitative assessment of internal bone surfaces. Structures such as vascular canals with a perimeter of 50 microns [19] can be identified under variable magnification. MBI based on SIE allowed for the identification of the quality of the localised areas of internal bone surface by the relative assessment of mineralised and unmineralised zones and vascular canals. Compared to the native extraction socket, the implant sites contain more unmineralised (22,19%) areas than extraction sockets (9,46%) (independent t test; p<0.05) and less vascular channels (0,58%) per surface area in relation to extraction sockets (1,81%) (Mann-Whitney Test; p<0.05). A summary of the results is shown in Fig 2A and 2B.

## Discussion

*In vivo* MBI has been used in animal experiments for more than 50 years, and different optical chambers have been developed and implanted, focusing on circulation and tissue growth in a well-defined titanium chamber. In clinical science, however, MBI in surgical disciplines has not been widely applied. The first reports of clinical endoscopic observations of bone in dental implant cavities were published more than 10 years ago [11]. MBI is different from contact endoscopy, which is used for the microscopic imaging of soft tissue surfaces where full contact with a high magnifying optical system is used. In contrast, SIE is carried out at a minimal distance from the bone surface. The tip of the scope is submerged and supported on a bone surface without being in complete contact. Thus, the irrigation fluid provides a transparent medium. MBI is performed at a short distance and thus produces a smaller optical magnification compared with contact endoscopy. In the case of the extraction sites, the socket itself represents a natural immersion chamber and can be used to provide short increments of jet flow for cleaning the field of view. If necessary, intraoral pressure monitoring may be applied [20] in the bone chamber to correlate the pressure data with the haemodynamics. This technique allows for the collection of a number of important haemodynamic measurements when used in conjunction with local and systemic arterial pressure data [8]. The natural surface of an extraction socket represents a prototype for MBI in artificial bone surfaces at any location in the skeleton, which may be created for diagnostic purposes. However, the natural “extraction socket” is a unique window for MBI in humans during the most frequent surgical intervention in medicine without provoking any additional trauma. Compared to implanted experimental bone chambers, SIE obviously cannot provide a repetitive evaluation at the identical bone site. However, the combination of SIE-based MBI with radiographic follow-up (cone beam CT and/or re-evaluation using marker implants) may serve as a baseline for a multimodal follow-up studies.

ImageJ is a well-known public domain image processing program that can be implemented using standard bone measurements, such as an ImageJ plugin, BoneJ, to make full use of the computer hardware [21]. However, for surface structure analysis, Image J 1.49m was used. In the future, different plugins may be used for the dynamic evaluation of blood flow. Software may be applied to more easily determine the area and number of vessels and non-mineralised spaces. Additional parameters may be measured using intravital staining [22] or fluorescence techniques [23].

### 5.1. Analysis of bone microstructure

The microstructure of the post-extraction cortical vascular area exhibited similar results as those presented by Kingsmill et al. (2007) [24], who identified cortical vascular canals with digital backscattered electron images. They found 3% of bone occupied by canals. Our measurement of the extraction sockets produced similar results. However, in the implant site sample, a smaller number of vascular channels was measured, which could be a consequence of artificial obstruction by drilling or anatomical factors. Dempster and Enlow (1959) [19] reported an average canal diameter of 30–50 microns; these canals are clearly visible in SIE images. The examples show that the image resolution of SIE is well above the average diameter of cortical canals, and therefore, it appears to be an adequate method for the evaluation of bone circulation.

The microstructure of bone in dental sites is mainly observed in the context of implant site classification (de Oliveira et al. (2012) [25] using the structural analysis of bone specimens (bone cores of implant cavities) obtained during the process of implantation. SIE allows the observation of bone surface and trabecular parameters directly at the surface of the cavity that later receives the implant; however, no images from inside the bone sample can be taken from the site. By instead measuring parameters at the adjacent surface within a biopsy bone volume, the measurement should more precisely describe the clinical situation before placing an implant because the biopsy volume does not represent the anatomical structure directly surrounding dental implants due to the tissue loss when using a trephine drill. Therefore, a systematic evaluation of dental implant sites by SIE-scanning and image analysis may be used in the future instead of evaluating of bone cores. The trabecular thickness (0,1 mm, [25]) and the number of trabeculae [26] can easily be detected using SIE. *In vivo* observation allows a complementary *in situ* view of the bone structure previously evaluated with 3D radiography.

In the present paper, mineralised and non-mineralised areas were differentiated, leading to the conclusion that the post-extraction alveolar surface mainly consists of a cortical layer with sparse non-mineralised zones. However, the observed implant sites demonstrated a higher degree of non-mineralised areas. Future observations may be carried out to obtain an *in vivo* implant-bone interface measurement before and after the placement of implants. BIC, which is obtained histologically or by using micro-CT or cone beam tomography, may be evaluated at the time of implant removal. Relative measurements of structures in a representative area are easily obtained using SIE, including the relative quantification of tissues, marrow spaces, trabeculae, vessels, and bone substitutes with reference to the area of interest. The limited depth of insight depends on the set of optical systems, surface distance and cavity diameter. Absolute measurements require a reference structure and a determination of the optical conditions. Calibration to determine the absolute values of tissue components is a study that should be carried out in the future.

### 5.2. Clinical application

Engelke and Galle (2008) [27] reported on 1568 SIE procedures in 595 patients to visualise routine implant cavities. Possible method-related complication, such as infection or intolerance of the method, were not observed, thereby providing evidence that SIE may be used routinely without major risk for the patients when using the window to the bone. Beltrán et al. (2012) [15] observed the type of bone density and its relation to vascular elements. This study provides an interesting method to assess newly formed bone in previously grafted bone areas, resting bone substitute particles and the number of nutritional vessels leading to the ability to quantitatively describe bone surface structures. In contrast to the rigid endoscopes used by our workgroup, Nahieli et al. (2011) [28] described the use of semiflexible 0.9-mm diameter endoscopes in implantology. This strategy may be successful in cavities with diameters below 3 mm; however, an important shortcoming is the relatively low resolution of the fiberscope’s images, which do not reach the resolution of rod lens optical systems.

Cone-Beam Computed Tomography (CBTC) has been commonly used to define the quality of alveolar bone [29–32], but the method is based on a subjective visual evaluation and thus still has some shortcomings for precise diagnosis and treatment with dental implants [29]. Direct contact imaging using *in vivo* MBI offers a complementary approach to determine the surrounding structure immediately before selecting and placing an adequate dental implant. However, MBI requires high resolution optical systems in bone cavities of sufficient diameter; a 2,7 or 4,0 mm optic may be used with an independent irrigation system. HD digital imaging improves the resolution.

The detection of soft tissue following vital staining using methylene blue is being used in periapical surgery and in contact endoscopy to obtain diagnostic information [22]. In the oral cavity, contact endoscopy allows for the histological evaluation of oral mucosa lesions [33]. Routine surgery of tooth extraction and bone cavity preparation during endosseous implantology appears to be a widely and commonly used opportunity to provide a window to the human bone structure without major discomfort due to the access.

With the development of imaging, vital staining and advanced optical tools, the new approach may be appreciated in dental as well as in medical diagnostics.

In the near future, SIE-based MBI could contribute valuable diagnostic information in bisphosphonate-related osteonecrosis of the jaw (BRONJ), which is a severe pathological entity in oral surgery. Using SIE/MBI, margins of the osteonecrosis may be determined under fluorescence guidance, as shown macroscopically by Pautke et al. (2010) [23]. SIE/MBI could serve as a powerful tool with which to observe tetracycline fluorescence on a microscopic base without opening the bone surface and without broad detachment of soft tissue. The technique might improve the precision of determining necrotic bone areas. Osteonecrosis following irradiation might be identified *in vivo* in the same manner via vascularisation or fluorescence under minimally invasive conditions. Using vital staining and local microscopic observation, SIE/MBI may also be used to detect a variety of bone pathologies.

## Conclusion

Using the post-extraction alveolus as a natural optical bone chamber, SIE/MBI allows for a morphometric *in vivo* evaluation at the microscopic level to observe and quantify the architecture and static and dynamic vascularisation of human internal osseous surfaces and opens a new diagnostic window for a large variety of evaluations.

## Supporting Information

S1 Movie Identification of vascular canals. Vascular canals were selected through presence of bleeding.
(AVI)
